# Supplementary material for: Effectiveness of a service design training program to improve clinical nurses’ compassion and problem-solving in Korea
Source: PLoS One. 2022 Aug 12;17(8):e0272957. doi: 10.1371/journal.pone.0272957 (PMC9374233; doi:10.1371/journal.pone.0272957)
Supplement: Attachment — Submitted filename: Comment forwarded by Getahun.docx [file pone.0272957.s001.docx]

**Title:** Implementing a Service Design Training Program to Improve Clinical Nurses’ Compassion and Problem-solving: A Mixed-Methods Pilot Study

**Comment 1**: It lacks specify of study area, period which makes it un attractive to some extent. Ho you see this issue?

Abstract: Dear Authors thank you for you work. Under this section the aim that you proposed is completely different from the title. As your title is about” *Implementing a Service Design Training Program to Improve Clinical Nurses’ Compassion and Problem-solving: A Mixed-Methods Pilot Study”* however you’re in your abstract it talks about specific program” line 28-30. The aim of this study was to examine the effects of a training program for patient experience-based nursing service design (PEN-SD) on clinical nurses’ compassion and problem”

**Comment 2**: How you can Negotiate these two different concepts here?

**Comment 3:** you have written the sentences on Line 37-38. “*This research is the first attempt to introduce a service design in the nursing field.”* Under result section of your abstract. How many data base you have searched to conclude the service design in nursing is first?

If you are sure and able to respond the above concern, please take the sentence to introduction section your abstract. If not, this sentences your personal saying which will not be scientifically sounding

**Comment 4:** Your abstract section lacks the method through which you passed to come up with the result that exist here. What will be your possible justification for this?

Generally I am not satisfied with the existing abstract unless it is improved.

**Comment 5:** Introduction line 51-53: is talking about Exploring patient engagement, however the title is about service design. As the introduction about first impression for reader it has to start with sentence talking about service design

**Comment 6:** line 59, 73 and 83 are you talking about Method or what you stated “Methodologies” which is not appropriate for your content.

**Comment 7**: line 72-88 were not cited elsewhere. How you will justify it?

**Comment 8:** result section was well organized however table and information with table were incomplete. i.e table are vague including title needs further improvement.

**Comment 9**: line 319 onward needs scientific evidence for possible justification.

**Comment 10:** 403-417/Conclusion of the document needs further improvement as it is not inline with the objective and finding of the study.

**Comment 11.** All references of the document needs further consideration for the format and to make the reference full.
