# Supplementary material for: Effectiveness of a service design training program to improve clinical nurses’ compassion and problem-solving in Korea
Source: PLoS One. 2022 Aug 12;17(8):e0272957. doi: 10.1371/journal.pone.0272957 (PMC9374233; doi:10.1371/journal.pone.0272957)
Supplement: Attachment — Submitted filename: Comment forwarded by Getahun 2nd.docx [file pone.0272957.s003.docx]

Comment 1: there are many improvements throughout the manuscript. However English language and grammar needs major revision still

Comment 2 Dear authors thank you for all your effort in revising your manuscript. However, I have a concern regarding the title and aim of the study. Do you think that they are the same?

“Evaluating a Service Design Training Program to Improve Clinical Nurses’ Compassion and Problem-solving in Korea” vs “examine the effects of the program on clinical nurses’ compassion and problem-solving ability”
