# Supplementary material for: Effectiveness of a service design training program to improve clinical nurses’ compassion and problem-solving in Korea
Source: PLoS One. 2022 Aug 12;17(8):e0272957. doi: 10.1371/journal.pone.0272957 (PMC9374233; doi:10.1371/journal.pone.0272957)
Supplement: Attachment — Submitted filename: Response lettter to Reviewer Comments.docx [file pone.0272957.s004.docx]

Thank you very much for your constructive feedback and the opportunity to revise our manuscript entitled “Effectiveness of a Service Design Training Program to Improve Clinical Nurses’ Compassion and Problem-solving in Korea” (PONE-D-21-21797R1). Your comments proved to be helpful for revising the manuscript. Below we described how we responded to your comments and highlighted the changes using the track changes mode in the manuscript. Thank you.

**Responses to Reviewer 1**

The manuscript needs a major English language edition. In another way, the manuscript title and aim were not consistent. I kindly recommend the authors make it consistent. There is a need for sentence re-arrangement in the introduction and abstract sections of the work.

Comment 1: there are many improvements throughout the manuscript. However, English language and grammar needs major revision still.

Response: Thank you. We had the revised manuscript proofread by a professional editor to improve the clarity of the manuscript. Revisions are track-changed in the manuscript.

Comment 2: Dear authors thank you for all your effort in revising your manuscript. However, I have a concern regarding the title and aim of the study. Do you think that they are the same?

“Evaluating a Service Design Training Program to Improve Clinical Nurses’ Compassion and Problem-solving in Korea” vs “examine the effects of the program on clinical nurses’ compassion and problem-solving ability”

Response: Following your recommendation, to make the title and aim consistent we revised the title as “Effectiveness of a Service Design Training Program to Improve Clinical Nurses’ Compassion and Problem-solving in Korea”. We think this title includes both implementing and evaluating of the program, and it is consistent with the aim: “The aim of this study was to implement a training program for patient experience-based nursing service design (PEN-SD) and examine the effects of the program on clinical nurses’ compassion and problem-solving ability.” (in the Abstract). Thank you for your understanding.

**Responses to Reviewer 2**

Almost all of the concerns raised in the previous revision are well addressed. However, further minor revisions are needed.

#1. As the study is an interventional study (before-after or quasi experimental) the word implementing on the title has no significance importance and the title can be written as “Effectiveness of a service design training program to improve clinical nurses’ compassion and problem-solving skill in Korea” or “Evaluating a service design training program to improve clinical nurses’ compassion and problem-solving skill in Korea”. In both titles implementation of the intervention is already there.

Response: As you recommended, we have now revised the title as “Effectiveness of a Service Design Training Program to Improve Clinical Nurses’ Compassion and Problem-solving in Korea”. This title indeed includes both implementing and evaluating of the program. Thank you.

#2. Under the participants heading. In line 100 you mentioned 21 participants were included in the study. in the same paragraph in line 108 the calculated sample size was 20. Why such discrepancies happened? In addition, though there is no means to calculate the sample size for qualitative study, “information saturation” as a basis to determine the sample size. However, nothing was said about it in the method section.

Response: We really appreciate your insightful feedback. The calculated sample size of 20 was the minimum number of the participants we needed. We also acknowledge that information saturation is a basis to determine the sample size in a qualitative study. Since we conducted structured interviews as the qualitative approach to support the initial quantitative findings, we collected data from the 21 nurses who participated in the program. Thank you for your understanding.

#3. Remove the bullets in lines 169, 182, 188, 195 and 200; simple separate the steps of the interventions in paragraphs.

Response: We have now removed the bullets and instead described each step of the interventions in paragraphs.

#4. Table and figure headings must be self-explanatory: in line 248 include the area, year and total sample size in bracket. line 167 too.

Response: We have now added the sample size information (*n* = 21) in the headings of all Tables and Figures. Thank you.

#5. In the discussion; information written from line 320-343 are more of explanation of the method section. Hence, better to minimize this information.

Response: Thanks to you, we realized that some information in the discussion was explaining the method part. Following your suggestion, we revised the discussion section as follows (pp. 17-18, Lines 328-354):

“In this study, a six-stage process for the PEN-SD training program was developed by adding the “Understand” and “Grow” phases to the existing four-stage process [20]. In the “Understand” phase—the first step of the program—participants selected the project topic, confirmed the matters to be solved during this training, and discussed the approach direction. It was a necessary phase for participants to establish the goals and plans of the project, before they actually discovered the problems. In future programs, it would be useful to organize the “Understand” phase based on the level of the participants so as to increase the effectiveness of the programs.

The “Grow” phase—the last stage of the PEN-SD training program—was originally designed to help participants apply and evaluate solutions derived through service design in the field, assess how to further develop them based on what they had learned, and suggest future activities [20]. However, in our pilot study applying the PEN-SD training program for a total of 24 hours, it was difficult to directly apply the project outcomes to the nursing clinical field. Future PEN-SD training programs require modifications so that participants can reflect and grow by directly applying the project outcomes to the field. Furthermore, it is imperative to keep the outcomes feasible in future programs with support from hospitals.”

**Responses to Reviewer 3**

All my concerns have been addressed by the authors and therefore have no further questions to raise. I recommend the revised manuscript for publication.

Response: Thank you very much.
