# Supplementary material for: Effectiveness of a service design training program to improve clinical nurses’ compassion and problem-solving in Korea
Source: PLoS One. 2022 Aug 12;17(8):e0272957. doi: 10.1371/journal.pone.0272957 (PMC9374233; doi:10.1371/journal.pone.0272957)
Supplement: Attachment — Submitted filename: Responses to Reviewer comments.docx [file pone.0272957.s006.docx]

**Responses to Reviewer Comments**

Thank you very much for your constructive feedback and the opportunity to revise our manuscript entitled “Effectiveness of a Service Design Training Program to Improve Clinical Nurses’ Compassion and Problem-solving in Korea” (PONE-D-21-21797R2). Your comments proved to be helpful for revising the manuscript. Below we described how we responded to your comments in blue and highlighted the changes using the track changes mode in the manuscript. Thank you.

**Overall observations**

The manuscript has improved a lot but it is still not in standard English. Secondly, I feel the discussion is heavy on the development of the program while giving less attention to assessment of effectiveness of the program. I suggest that the authors try to strike a balance or alternatively drop the development bit and only deal with assessing the effectiveness in this manuscript. If they take this route they can develop another manuscript to take care of the development bit. That way this manuscript will be clearer. To support this point, you can see that in the results section there is hardly anything about development of the program, which makes it strange then when the discussion is heavy with development. Further, by describing development of the program in the methods section, it already looks like the intention of the authors in this manuscript was to report on the effectiveness of the program only.

We really appreciate your thorough review and helpful suggestions to improve clarity of the manuscript. Following your suggestions, we have now deleted the development part in Discussion in particular, and revised some English expressions. Thank you.

**Specific edits and comments**

Line 29 & 30: objective may have to be amended if authors decide to drop the development/implementing bit

We revised the aim of this study as follows (p. 2, Lines 28-31):

“The aim of this study was to examine the effects of a training program for patient experience-based nursing service design (PEN-SD) on clinical nurses’ compassion and problem-solving ability.”

Line 36: Delete “in terms of”

Line 36-37: Consider rephrasing to read as follows: “In the structured interviews, majority of participants (n=17) felt that they were able to consider problems from patient’s perspective”

Line 39: Delete “This research is an innovative attempt to introduce a service design in the nursing field” because its not supported in the results

Line 41-42: Delete “as a service development method to provide the best nursing services reflecting patients’ experiences”

Line 55: replace “fresh” with “new”

Line 57: Rephrase “design has been recently applied” to “has recently been applied”

Line 63-64: Delete “and a support program required for the treatment process”

According to your comments, we have edited all the parts you mentioned above.

Line 66-68: For example, a radiology department benefited from service design by reducing the waiting period for examinations (from xx to xx) and increasing the number of examinations (from xx to xx) as well as revisits to the hospital.

We provided more information as follows (p. 3, Lines 66-68):

“For example, a radiology department benefited from service design by reducing the waiting period for examinations from 2.6 days to 1.5 days and increasing the number of examinations from 21,078 to 22,236 as well as revisits to the hospital [10].”

Line 70-71: delete “extremely” and “patients’ satisfaction with”

Line 75: Delete “the field of”

Line 76-78: Rephrase “it is imperative that those nurses who are most in contact with patients should empathize with the patient’s experience and reduce problems related to nursing services.” To read “it is imperative that nurses, who spend most most of their time with patients empathize with the patient’s experience and develop problem-solving skills.”

Line 78-80: Rephrase “Additionally, service design utilizes different instrumental methods step by step, which necessitates a systematic training program [1,2].” To “service design utilizes different methods in a step-wise approach which necessitates a systematic training program”

Line 80-82: rephrase “To the best of our knowledge, no research has yet been conducted on 81 the development of a nursing service design training program to nurture service design 82 capabilities in nurses.” To read “To the best of our knowledge, no research has been conducted to assess the effectiveness of a nursing service design training program to improve nurses compassion and problem solving in Korea”

Line 85: Add “we hypothesize that” the patient experience-based nursing service design (PEN-SD)….

Line 87-88: rephrase “Therefore, we developed this training program for nurses and verified how it influenced 88 their compassion and problem-solving ability.” To read “Therefore, we developed this training program and assessed its effectiveness in improving compassion and problem-solving ability.”

Line 93-94: Add “improving” between “on” and “nurses” so that it reads….”on improving nurses compassion…”

Line 107: replace “verified” with “assessed”

On the section where you describe participants i.e line 99-111, bring up line 104-111 to be the first paragraph while line 100-104 becomes the second paragraph. With other edits made in the text, the section should then read as below:

The sample size was calculated using the G*power 3.1.9 program. In previous research, where the effectiveness of the program in improving nurses’ compassion and problem-solving ability has been assessed [15, 16], the effect size was calculated per variable, which was 0.5–0.6. The effect size for the current research was based on the aforementioned result. The minimum sample size was 20, calculated for analysis using the Wilcoxon signed-rank test based on the aforesaid result with the power of the test (1-β) = 0.8, effect size (d) = 0.6, and significance level (α) = .05.

We conveniently sampled 25 participants out of whom only 21 were included in the analysis, after excluding four who responded poorly to the surveys. The final participants—21 nurses working at a university hospital in Korea— were either charge nurses or held positions high enough to influence changes in nursing services. They understood the purpose of the research and were willing to participate in five weeks of training.

Line 116: Use “where’ instead of “wherein” and revise through and through

Line 117: replace “in this study” with “in the current study”

Line 124-125: rephrase the question to read as follows “are there any differences after the training in terms of patient care related problems that can occur in the field?

Line 126: Add “the” between “of” and “program”

Line 126-127: Not clear

Line 131-135: Difficult to understand. Do you mean to say: We developed the PEN-SD training program based on the four-stage double diamond process by adding the “understand” and “grow” phases from the six-stage service design [20].

Line 255-258: Rephrase to read as follows” “the results of each sub scale was as follows: communication (Z = −2.42, p = .016), sensitivity (Z = −1.41, p = .159) and insight (Z = −0.51, p = .608) indicating a significant improvement in communication but not in sensitivity and insight.

Line 259-260: rephrase to read “ there was no significant differences in problem solving before and after the training (median scores 3.73 and 3.82 [Z = −1.01, p = .313]) respectively.

According to your comments, we have edited all the parts you mentioned above.

Line 318-333 and throughout the discussion: why emphasize on development of the program yet the study is about assessing effectiveness of the program? Further you hardly report anything about development in your results!

Thank you for your insightful feedback. We have now deleted the first and second paragraphs on development in Discussion.
